# Influence of seminal cryopreservation on embryo kinetics. Image analysis and clinical outcomes

**DOI:** 10.5935/1518-0557.20260005

**Published:** 2026

**Authors:** MC Lorenzo Rodríguez, M Martínez Morales, I Pérez Cano, P Hernández Vargas, B Gadea Navarro, M Roldán Ramírez, B. Amorocho Llanos, A. Doménech Vidal, MA Valera Cerdá, M Muñoz Cantero, M. Meseguer Escrivá

**Affiliations:** 1 IVF Laboratory, IVIRMA Alicante, Alicante, Spain; 2 IVIRMA Foundation, Valencia, Spain; 3 Reproductive Unit, IVIRMA Alicante, Alicante, Spain; 4 IVF Laboratory, IVIRMA Valencia, Valencia, Spain

**Keywords:** sperm, cryopreserved, morphokinetic, embryo development, clinical outcome

## Abstract

**Objective:**

This study aims to compare the effect of using fresh ejaculated sperm versus cryopreserved sperm on the morphology and kinetics of embryo development and on clinical reproductive outcomes.

**Methods:**

Retrospective, observational, single-centre study conducted at IVIRMA Alicante (June 2013-December 2020) that included 469 oocyte donation cycles with frozen, fresh and mixed eggs in which ICSI, IVF or IVF-ICSI with fresh ejaculated and frozen ejaculated partner sperm was performed. For each group, the morphokinetic variables t2, t3, t4, t5, t6, t7, t8, tM, tSB, tB, tEB were studied in addition to the clinical pregnancy rate, implantation rate, ongoing pregnancy rate, miscarriage rate, and live birth rate, per transfer and per embryo transferred. Statistical analysis was performed using the IBM SPSS^®^ software. Values of *p*≤0.05 were considered statistically significant.

**Results:**

Longer development times were observed in the fresh semen group, with significantly longer times for t2, t3, t4, t5, tB, tEB. Clinical results per transfer showed a trend, although not significant, towards lower rates in the cryopreserved semen group compared to the fresh semen group. However, the miscarriage rate was higher for the cryopreserved semen group, although this difference was not statistically significant.

**Conclusions:**

Our results indicate that embryos from fresh semen have a slower developmental rate for the morphokinetic parameters studied. Since no significant differences in clinical reproductive outcomes were found, it is concluded that sperm freezing is a valuable technique that can continue to be applied. However, further studies are needed to confirm these results.

## INTRODUCTION

The term ‘cryopreservation’ refers to the cooling of cells and tissues to very low temperatures in order to stop all biological activity and preserve them intact for future use (Practice Committees of the American Society for Reproductive Medicine and the Society for Assisted Reproductive Technology, 2013)

Today, gamete and embryo cryopreservation has become worldwide as the key technique in assisted reproduction clinics, due to the many advantages it offers. However, as was to be expected, seminal cryopreservation was the first gamete freezing technique to be developed. So much so that the first record of human sperm cryopreservation dates back to approximately 1776 (Spallanzani, 1776). After this time, other studies were carried out (Jahnel, 1938). The first scientific progress related to sperm cryopreservation came from Polge *et al.* (1949) when, by making use of the cryoprotective properties of glycerol, they achieved greater sperm survival and motility after thawing. This breakthrough marked a turning point in the field of fertility preservation, which has continued to develop to the present day.

Compared to other cell types, spermatozoa tend to be less sensitive to freezing damage, mainly due to their high membrane fluidity and low water content (50%). Even so, cryopreservation produces deleterious effects (related to osmotic variations and mechanical breakage) that cause changes to the structure and function of spermatozoa that must be taken into account (Di Santo *et al.*, 2012). Among the negative effects of seminal cryopreservation on spermatozoa are those affecting the composition and functionality of the plasma membrane. Specifically, cryopreservation affects the carbohydrates that make up the membrane glycocalyx (Talaei *et al.*, 2010), which impairs the function of membrane proteins bound to it, which are responsible for ion transport and metabolism, thus affecting fertilization capacity (Diekman, 2003; Di Santo *et al.*, 2012).

Likewise, uncontrolled water entry into spermatozoa can change cell osmolality and deform the membrane structure, which could consequently affect sperm morphology (O’Connell *et al.*, 2002; Ozkavukcu *et al.*, 2008) and lead to head loss as well as tail defects (Hezavehei *et al.*, 2018). In addition, some authors have indicated that freezing/thawing causes damage to the acrosomal membrane, disintegrating it and depleting its acrosomal content, which is indispensable for gamete interaction to take place (Barthelemy *et al.*, 1990), and even lower expression of certain acrosome-related proteins after thawing has been reported (Bogle *et al.*, 2017; Hezavehei *et al.*, 2018).

Sperm freezing can also cause oxidative stress, which induces the production of ROS (reactive oxygen species) and decreases the levels of antioxidant factors, leading to lipid peroxidation, deoxyribonucleic acid (DNA) fragmentation, apoptosis and membrane damage (mitochondrial and cytoplasmic) after thawing (Toro *et al.*, 2009; Gualtieri *et al.*, 2021). Regarding possible DNA damage, some studies have reported significant alterations in sperm DNA integrity after cryopreservation (Donnelly *et al.*, 2001; de Paula *et al.*, 2006) or even alterations in transcription and interactions of messenger ribonucleic acid (mRNA) with proteins in spermatozoa, which can directly influence embryonic development (Valcarce *et al.*, 2013).

With all that has been said so far, it has been demonstrated by a multitude of studies and research that sperm cryopreservation can cause changes in sperm physiology and sperm quality parameters. However, very few studies have focused on whether this effect influences embryo development and reproductive outcomes. Moreover, most of them evaluate embryo quality based on morphological criteria of embryo selection.

Therefore, the main objective of this study is to analyze the influence of seminal cryopreservation on embryo quality as assessed by their developmental kinetics and to establish a relationship between these factors and the clinical reproductive outcomes.

## MATERIAL AND METHODS

### Study design and eligibility criteria

Retrospective, observational, single-center study performed at IVIRMA Alicante, Alicante (Spain). The study included 469 cycles of patients who were part of the oocyte donation program between June 2013 and December 2020, undergoing ICSI or split IVF-ICSI cycles. All patients with a body mass index (BMI) <30 kg/m^2^ and age between 18-50 years old were included. All women with uterine or systemic pathology that could affect the outcome of the cycle were excluded. As for the semen samples, all fresh ejaculate samples with at least 10% progressive motile spermatozoa were included. In the frozen semen group, all semen samples with motility greater than 10% progressive motile spermatozoa before freezing and greater than 5% after thawing were included. All severe oligoasthenoteratozoospermic semen samples according to WHO criteria (World Health Organization, 2010), banked semen and semen samples processed by Magnetic-Activated Cell Sorting (MACS) were excluded.

### Clinical protocol

All oocyte donors underwent controlled ovarian stimulation with gonadotropin-releasing hormone (GnRH) antagonist and progestogen treatment protocols. When an adequate follicular response was obtained, ovulation was induced with GnRH agonists and oocytes were retrieved by ultrasound-guided follicular puncture 36±2 hours after GnRH agonist administration.

### Preparation of semen samples

Semen samples included in the study were frozen by applying the freeze-pill protocol (using a dry ice plate) with SpermFreeze™ cryoprotectant (FertiPro^®^; Beernem, Belgium) or cryoprotectant Refrigeration Medium - Test Yolk Buffer (TYB) with Gentamicin (FUJIFILM^®^ Irvine Scientific; Santa Ana, California, USA) (Meseguer *et al.*, 2007).

Semen samples were thawed (37°C for 20 minutes) or collected in a sterile container by masturbation on the day of IVF or ICSI. On the one hand, thawed semen for ICSI or IVF was capacitated by density gradients followed by swim-up. On the other hand, fresh semen was capacitated by density gradients plus swim-up in split IVF/ICSI samples, and only swim-up in ICSI samples.

For this purpose, the following media were used ALLGrad^®^ 100%, ALLGrad Wash^®^ and global^®^ total^®^ for Fertilization (LifeGlobal^®^; Connecticut, USA) or Sil-Select STOCK™ and FertiCult™ Flushing medium (FertiPro^®^; Beernem, Belgium).

### In vitro culture: fertilisation and embryo development

The oocytes obtained were fertilised (by IVF or ICSI) and placed in pre-equilibrated embryo culture plates (Embryoslides, Unisense Fertilitech; Aarhus, Denmark) with global^®^ total^®^ (LifeGlobal^®^; Connecticut, USA) or Sequential Cleav™ (ORIGIO^®^; Målov, Denmark) embryo culture media and covered with mineral oil (LiteOil^®^ (LifeGlobal^®^; Connecticut, USA) or Hypure™ Oil (Kitazato^®^; Yanagishima, Fuji, Shizuoka, Japan). Embryos were cultured to blastocyst stage with global^®^ total^®^ embryo culture media (LifeGlobal^®^; Connecticut, USA) or Sequential Blast™ (ORIGIO^®^; Målov, Denmark) in the Embryoscope™ time lapse incubator (Vitrolife, Sweden) at 37°C, 6-7% CO_2_ and under reduced oxygen pressure (5% O_2_).

### Embryo transfer

The kinetics of embryo division was studied using EmbryoScope™ digital image capture and the series of images collected were analysed in Embryoviewer® (Vitrolife). In addition, morphological assessment was performed according to the morphological classification system of the Association for the Study of Reproductive Biology (ASEBIR) on day 2 and 3, as well as on day 5 and 6 post-fertilisation (ASEBIR, 2015).

### Statistical analysis

The morphokinetic parameters of each embryo were collected, recorded and exported from Embryoviewer® software (Vitrolife) (Herrero *et al.*, 2013). Likewise, the clinical reproductive variables of all cycles were exported from the database.

Cross-tabulations were performed to compare the distribution of confounding variables between the two study groups (frozen and fresh semen), using the chi-square test (χ^2^) for categorical confounding variables (insemination technique, seminal diagnosis and oocyte type) and the ANOVA test for the continuous confounding variables (age of the oocyte, age of the patient, BMI of the patient, volume, concentration, motility of fresh and capacitated semen, BMI and age of the male, number of oocytes, number of MII oocytes, number of fertilized oocytes and fertilization rate).

The analysis of morphokinetic parameters was performed by comparing means and standard deviation (SD) of each of the morphokinetic variables described in Table 1, for each study group (frozen semen and fresh semen), applying the ANOVA statistical test. The association of these parameters with the study groups was also analyzed using a logistic regression model. In addition, the effect of each confounding variable on each of the morphokinetic parameters was studied by applying a linear multivariate model.

**Table 1:** Nomenclature of the morphokinetic parameters studied. Description of the parameters obtained from ESHRE Working group on Time-lapse technology *et al*. (2020).

Morphokinetics parameters	Description
**tPB2**	The second polar body is completely detached from the oolemma (h)
**TPNa**	Appearance of individual pronuclei; tPN1a, tPN2a, tPN3a, ... (h)
**TPNf**	Time frame of pronuclei fading; tPN1f; tPN2f... (h)
**t2**	Embryonic division time from fertilization to 2 cells (h)
**t3**	Embryonic division time from fertilization to 3 cells (h)
**t4**	Embryonic division time from fertilization to 4 cells (h)
**t5**	Embryonic division time from fertilization to 5 cells (h)
**t6**	Embryonic division time from fertilization to 6 cells (h)
**t7**	Embryonic division time from fertilization to 7 cells (h)
**t8**	Embryonic division time from fertilization to 8 cells (h)
**t9+**	Embryonic division time from fertilization to 9 cells (h)
**tSC**	First evidence of compaction (h)
**tM**	Time of completion of compaction process (h)
**tSB**	Initiation of blastulation (first frame in which the blastocoel is visible)(h)
**tB**	Full blastocyst (last frame before zona starts to thin) (h)
**tEB**	Time of expanded blastocyst formation (h)
**tHB**	Fully hatched blastocyst (h)

The embryo development rate at day 3 and day 5 was also compared between the fresh and frozen semen groups, using the ANOVA statistical test for the comparison of means. Embryo quality was also analyzed using the chi-square test, stratifying by confounding variables.

Finally, the evaluation of clinical outcomes (clinical pregnancy rate, implantation rate, ongoing pregnancy rate, miscarriage rate and live birth rate (LBR)) per transfer was carried out using the chi-square test for categorical variables and applying the ANOVA test for continuous variables (implantation rate after single or double transfer). Clinical outcomes per embryo transferred were analyzed using a logistic regression model. Statistical analysis was performed with IBM SPSS^®^ software. Values of *p*≤0.05 were considered to denote a significant difference.

## RESULTS

The study included 469 cycles of which 266 were performed with fresh ejaculated semen and 203 with frozen ejaculated semen. The ICSI technique was used in 398 cycles, while in the remaining cycles conventional in vitro fertilization IVF (9) and split IVF-ICSI (62) were performed. Regarding the type of oocyte, fresh oocytes were used in 186 cycles, vitrified oocytes in 272 cycles and mixed oocytes in the remaining 11 cycles. Finally, a total of 4426 correctly fertilized oocytes were obtained, of which 2,523 corresponded to the fresh sperm group and 1,903 to the frozen sperm group.

There were significant differences (*p* value=0.006) in terms of the insemination technique used and the sperm group (fresh or frozen), such that the number of cycles in which ICSI was used was higher than the number of cycles in which IVF-ICSI and IVF were applied, both in the fresh semen group (ICSI=216 (81.2%), IVF-ICSI=41 (15.4%), IVF=9 (3.4%)) and in the frozen sperm group (ICSI=182 (89.7%), IVF-ICSI=21 (10.3%), IVF=0 (0%)).

In addition, the number of cycles using normozoospermic semen was significantly higher (*p* value=0.005) than the number of cycles using oligozoospermic samples, both for fresh semen (196 (73.7%), 39 (14.7%)) and frozen semen (168 (82.8%), 11 (5.4%)), respectively. It should be noted that 55 cycles had no semen analysis value.

With regard to the type of oocyte (fresh, vitrified and mixed), no significant differences were found (*p* value=0.734) in terms of the use of one type of oocyte or another and the status of the semen (fresh or frozen) (Table 2).

**Table 2:** Crosstabulation table comparing the distribution of confounding variables between the fresh and frozen semen groups.

VARIABLE	CATEGORY	FRESH SPERM (N=266)	FROZEN SPERM (N=203)	TOTAL (469 CYCLES)	p
**INSEMINATION** **TECHNIQUE**	IVF	9 (3.4)	0	9 (1.9)	**0.006**
	IVF/ICSI	41 (15.4)	21 (10.3)	62 (13.2)	
	ICSI	216 (81.2)	182 (89.7)	398 (84.9)	
**SEMINAL DIAGNOSIS**	Normospermia	196 (73.7)	168 (82.8)	364 (77.6)	**0.005**
	Oligozoospermia	39 (14.7)	11 (5.4)	50 (10.7)	
	NA	31 (11.7)	24 (11.8)	55 (11.7)	
**OOCYTE TYPE**	Fresh	107 (40.2)	79 (38.9)	186 (39.7)	0.734
	Mixed	5 (1.9)	6 (3)	11 (2.3)	
	Vitrified	154 (57.9)	118 (58.1)	272 (58)	

* The number of cycles for each group and the percentage (%) is indicated in parentheses. NA: Not applicable. The significance level has been considered to be 5%, so *p* value<0.05 is considered significant.

When analyzing the demographic data of the female partner, no significant differences were found for patient age, BMI and oocyte age between the fresh and frozen sperm groups. Likewise, for the male partner, no significant differences were found for age (*p* value=0.935), but significant differences were found for BMI value (*p* value=0.040), being higher for the fresh semen group (26.63; 95%CI:26.19-27.08), than for the frozen semen group (25.95; 95%CI:25.49-26.41).

Similarly, significant differences were found for sperm volume (*p* value=0.000), fresh concentration (*p* value=0.000), percentage of progressive spermatozoa in fresh (*p* value=0.000) and volume after capacitation (*p* value=0.000). For the rest of the sperm values, as for the oocyte characteristics, no significant differences were found between the fresh and frozen semen groups (Table 3).

**Table 3:** Comparison of continuous confounding variables between the two study groups.

		FRESH SPERM (N=266)	FROZEN SPERM (N=203)	p
**CYCLES**		266	203	
**FEMALE CHARACTERISTICS**	Age (years old)	41.43 (40.96-41.90)	41.39 (40.82-41.96)	0.916
	BMI (kg/m^2^)	22.89 (22.54-23.25)	23.03 (22.62-23.45)	0.609
	Oocyte age (years)	26.98 (26.0-27.90)	26.03 (25,17-26.90)	0.147
**MALE CHARACTERISTICS**	Age (years old)	42.35 (41.55-43.15)	42.40 (41,53-43.27)	0.935
	BMI (kg/m^2^)	26.63 (26.19-27.08)	25.95 (25.49-26.41)	**0.040**
**SEMINAL CHARACTERISTICS**	Fresh seminal volume (ml)	3.25 (2.84-3.66)	0.96 (0.91-1.02)	**0.000**
	Fresh seminal concentration (mill/ml)	51.60 (46.92-56.28)	70.11 (62.18-78.04)	**0.000**
	Fresh progressive spermatozoa (%)	42.66 (40.28-45.04)	19.17 (17.69-20.65)	**0.000**
	Seminal volume capacitated (ml)	0.22 (0.20-0.26)	0.14 (0.12-0.16)	**0.000**
	Concentration post work up (mill/ml)	1.97 (0.97-2.98)	1.74 (0.39-3.10)	0.784
	Progressive spermatozoa post work up (%)	94.32 (91.98-96.67)	94.05 (91.63-96.47)	0.875
**OOCYTE CHARACTERISTICS**	Nº of oocytes recovered/cycle	13.06 (12.82-13.31)	12.91 (12.66-13.16)	0.384
	Nº of oocytes MII/cycle	11.49 (11.07-11.91)	11.92 (11.56-12.29)	0.143
	Nº of oocytes inseminated/cycle	12.45 (12.21-12.68)	12.21 (11.95-12.47)	0.190
	Nº of oocytes fertilized/cycle	9.48 (9.20-9.77)	9.37 (9.08-9.67)	0.602
	Fertilization rate (%)	76.12 (74.39-77.86)	77.03 (75.07-78.99)	0.497

* Results are presented as mean (95% CI). The significance level has been considered to be 5%, so *p* value<0.05 is considered significant.

### Embryo development and embryo quality

Although the number of embryos that developed to day 3 and day 5 was higher for the fresh semen group, there were no significant differences in embryo development at day 3 (*p* value=0.501) and blastocyst formation rate (day 5) (*p* value=0.682) between the two groups (Table 4). The percentage of viable embryos (transferred or frozen embryos) was higher when fresh semen was used, although the differences were not significant between the two groups, both overall (*p* value=0.094) and considering confounding variables.

**Table 4:** Proportion of embryos that reach day 3 and proportion of embryos that develop to blastocyst, by cycle and for the study groups.

		Mean (95% IC)	p
**D3 RATE (%)**	Fresh sperm	98.05 (97.38-98.72)	0.501
	Frozen sperm	97.69 (96.87-98.51)	
	Total	97.90 (97.38-98.41)	
**BLASTOCYST RATE (%)**	Fresh sperm	68.68 (65.73-71.63)	0.682
	Frozen sperm	67.76 (64.51-71.01)	
	Total	68.28 (66.10-70.46)	

* Development rate on D3: Proportion of embryos that reach day 3 with respect to correctly fertilized oocytes. Blastocyst rate: proportion of embryos that develop to blastocyst with respect to viable embryos on day 3. The results are presented as a percentage (95% CI). The significance level was considered to be 5%, so *p* value< 0.05 is considered significant.

Significant differences were only found for the mixed oocyte group, where the percentage of viable embryos was higher for the fresh semen group than for the frozen group (49.1%, 25.6%, *p* value=0.019).

### Morphokinetic parameters of embryo development

When the morphokinetic variables were studied in the 2 study groups, shorter times were observed in the frozen semen group for all the morphokinetic parameters studied, being significantly shorter for the variables t2, t3, t4, t5, tB, tEB (Table 5).

**Table 5:** Time division in hours of the morphokinetic parameters obtained for each study group.

	FRESH SPERM (N=2523)	FROZEN SEMEN (N=1903)	p
**t2**	27.48 (27.24-27.71)	26.83 (26.58-27.07)	0.000
**t3**	37.78 (37.50-38.05)	37.20 (36.92-37.48)	0.005
**t4**	40.40 (40.07-40.72)	39.53 (39.22-39.85)	0.000
**t5**	51.04 (50.64-51.44)	50.23 (49.81-50.65)	0.007
**t6**	54.25 (53.84-54.65)	53.71 (53.24-54.17)	0.088
**t7**	57.57 (57.11-58.02)	56.95 (56.42-57.48)	0.083
**t8**	61.51 (61.00-62.03)	61.13 (60.52-61.74)	0.348
**tM**	87.75 (87.31-88.20)	87.50 (86.96-88.04)	0.471
**tSB**	99.18 (98.78-99.58)	98.62 (98.15-99.09)	0.078
**tB**	104.50 (104.10-104.91)	103.52 (103.04-103.99)	0.002
**tEB**	110.82 (110.35-111.29)	109.47 (108.97-109.98)	0.000

* The results are presented as mean (95% CI) and the comparison was made per embryo. The significance level has been considered to be 5%, so *p* value<0.05 is considered significant.

**Table 5:** Time division in hours of the morphokinetic parameters obtained for each study group.

	FRESH SPERM (N=2523)	FROZEN SEMEN (N=1903)	p
**t2**	27.48 (27.24-27.71)	26.83 (26.58-27.07)	0.000
**t3**	37.78 (37.50-38.05)	37.20 (36.92-37.48)	0.005
**t4**	40.40 (40.07-40.72)	39.53 (39.22-39.85)	0.000
**t5**	51.04 (50.64-51.44)	50.23 (49.81-50.65)	0.007
**t6**	54.25 (53.84-54.65)	53.71 (53.24-54.17)	0.088
**t7**	57.57 (57.11-58.02)	56.95 (56.42-57.48)	0.083
**t8**	61.51 (61.00-62.03)	61.13 (60.52-61.74)	0.348
**tM**	87.75 (87.31-88.20)	87.50 (86.96-88.04)	0.471
**tSB**	99.18 (98.78-99.58)	98.62 (98.15-99.09)	0.078
**tB**	104.50 (104.10-104.91)	103.52 (103.04-103.99)	0.002
**tEB**	110.82 (110.35-111.29)	109.47 (108.97-109.98)	0.000

* The results are presented as mean (95% CI) and the comparison was made per embryo. The significance level has been considered to be 5%, so *p* value<0.05 is considered significant.

After stratified analysis according to the confounding variables, significant differences were also found between the fresh and frozen semen groups for all the morphokinetic variables shown in Table 6.

**Table 6:** Time division in hours of the morphokinetic parameters that were significant for each confounding variable and study group.

		MORPHOKINETIC PARAMETER	FRESH SPERM (N=2523)	FROZEN SPERM (N=1903)	p
**INSEMINATION TECHNIQUE**	IVF-ICSI	tSB tB tEB	100.23 (99.20-101.25) 105.28 (104.25-106.32) 111.94 (110.63-113.25)	97.94 (96.56-99.31) 102.58 (101.11-104.04) 109.24 (107.65-110.82)	0.01 0.003 0.011
	ICSI	t2 t3 t4 t5 tEB	27.48 (27.23-27.73) 37.77 (37.46-38.07) 40.43 (40.06-40.79) 51.07 (50.61-51.52) 110.42 (109.93-110.91)	26.80 (26.55-27.06) 37.21 (36.91-37.51) 39.48 (39.15-39.81) 50.15 (49.71-50.59) 109.50 (108.97-110.04)	0.000 0.012 0.000 0.005 0.013
**OOCYTE TYPE**	Fresh	t2 t3 t4 t5 t6 t7 t8	27.17 (26.82-27.52) 37.50 (37.11-37.90) 39.81 (39.37-40.26) 50.78 (50.19-51.38) 54.36 (53.74-54.97) 57.49 (56.83-58.15) 61.62 (60.85-62.38)	26.32 (25.98-26.67) 36.73 (36.32-37.14) 38.86 (38.40-39.31) 49.62 (48.99-50.26) 53.00 (52.29-53.70) 56.17 (55.38-56.96) 60.17 (59.27-61.07)	0.001 0.009 0.004 0.010 0.005 0.011 0.016
	Vitrified	t2 t4 tB tEB	27.73 (27.42-28.05) 40.85 (40.38-41.31) 104.55 (104.03-105.07) 110.78 (110.15-111.40)	27.22 (26.89-27.54) 40.01 (39.57-40.44) 103.46 (102.82-104.10) 109.19 (108.51-109.87)	0.027 0.011 0.009 0.001
	Mixed	tM	92.92 (90.73-95.12)	88.17 (84.95-91.40)	0.015
**SEMINAL** **DIAGNOSIS**	Normospermia	t2 t3 t4 t5 tSB tB tEB	27.74 (27.45-28.02) 38.06 (37.73-38.38) 40.80 (40.41-41.19) 51.58 (51.10-52.05) 99.70 (99.23-100.18) 105.05 (104.57-105.53) 111.39 (110.82-111.96)	26.95 (26.69-27.21) 37.36 (37.06-37.67) 39.71 (39.37-40.04) 50.62 (50.15-51.09) 98.84 (98.32-99.36) 103.77 (103.2-104.29) 109.82 (109.25-110.38)	0.000 0.003 0.000 0.005 0.016 0.000 0.000
	Oligozoospermia	tM	85.82 (84.69-86.95)	88.69 (86.46-90.93)	0.017

* The results are presented as mean (95% CI) and the comparison was made per embryo. The significance level has been considered to be 5%, so *p* value<0.05 is considered significant.

A significant association was also reported between the morphokinetic parameters t2 (*p* value=0.009, OR=0.921) and tEB (*p* value=0.016, OR=0.964) with the frozen semen group, so that, for these variables, shorter times are presented in the frozen semen group than in the fresh semen group (the higher the morphokinetic values, the lower the probability of belonging to the frozen semen group) (data not shown in the results tables).

When analyzing the effect of each variable on outcome independently, we found that the vitrified oocyte group was associated with higher values of t2 (*p* value=0.000), t3 (*p* value=0.009), t4 (*p* value=0.000), t5 (*p* value=0.024), t8 (*p* value=0.028), tM (*p* value=0.000) and tSB (*p* value=0.013) compared to fresh oocyte group (data not shown in the results tables).

### Clinical outcomes per transfer and per embryo transferred

There were no significant differences in clinical pregnancy rate (*p* value=0.714), implantation rate (*p* value=0.562), ongoing pregnancy rate (*p* value=0.237) and LBR (*p* value=0.388); although there was a tendency to obtain higher rates in the fresh semen group (Table 7).

**Table 7:** Clinical results by transfer in the different study groups.

	FRESH SPERM	FROZEN SPERM	p value
**CLINICAL GESTATION (%)**	53.8	52.0	0.714
**IMPLANTATION RATE (%)**	50.6 (44.69-56.49)	48.0 (41.36-54.60)	0.562
**ONGOING PREGNANCY RATE (%)**	45.5	39.9	0.237
**MISCARRIAGE RATE (%)**	29.0	33.1	0.469
**LBR (<=1) (%)**	43.7	39.6	0.388

* The results are presented as mean (95% CI) or proportion. The significance level was considered to be 5%, so that a *p* value<0.05 is considered significant. The chi-square statistical test was performed for categorical variables and T Student (ANOVA) for continuous variables (implantation rate).

†- Clinical gestation rate (%): Proportion of pregnancies in relation to the total number of transfer cycles, determined by the presence of at least one embryo with positive cardiac activity on ultrasound at 5-6 weeks after oocyte retrieval. Implantation rate (%): Ratio between the number of gestational sacs visible sonographically and the number of embryos transferred. Ongoing pregnancy rate (%): Proportion of clinical gestations that do not end in clinical abortion or ectopic pregnancy. Miscarriage rate (%): Number of gestational losses in relation to total pregnancies. LBR (%): Proportion of live newborns in relation to cycles performed.

There were no significant differences when stratifying the results by confounding variables. Binary logistic regression analysis showed no significant impact of the effect of study variables and confounding variables on clinical outcomes.

When analyzing the clinical outcomes per embryo transferred and being able to consider the variables relating to each embryo; again, there were no significant differences when comparing the fresh and frozen semen group for implantation rate (*p* value=0.833), LBR (*p* value=0.704) and miscarriage rate per embryo transferred (*p* value=0.456).

However, significant differences were found for the morphokinetic parameter tB (*p* value=0.007, OR=1.173) when studying the implantation rate per embryo transferred. This result reflected that, by increasing the tB value by one hour, embryos have a statistically significant 1.173 times higher probability of implantation.

## DISCUSSION

The main objective of this study was to evaluate the influence of semen freezing on embryo morphokinetic parameters. The results of this study suggest that the use of frozen semen could affect early embryo development in terms of morphokinetic parameters, finding significantly shorter times in the frozen semen group for the parameters t2, t3, t4, t5, tB, tEB compared to the fresh semen group. These results differ from the data published by Eastick *et al.* (2017), where no significant differences (*p* value<0.05) were found in the timing of any of the key embryonic developmental events (PN_t1, nuclear envelope breakdown (NEBD), cytokinesis, t2, t3, t4, t5, t6, t7, t8, tM, tSB, tEB, tHB, s1, s2, s3, cc2 and cc3) when using fresh ejaculated sperm or cryopreserved sperm in ICSI cycles. However, the rates of development up to day 3 and 5 were comparable between the two studies, with no significant difference between the fresh and frozen semen groups (Eastick *et al.*, 2017).

Another similar study is that of Hickman *et al.* (2013). In it, embryos derived from frozen spermatozoa from testicular biopsy reached the two-cell stage significantly earlier (28.5±5.0 *vs*. 26.9±3. 9 hours post injection (hpi), *p*=0.03), while morula formation (93±12 *vs*. 97±13hpi, *p*=0.045) and blastocyst hatching (119±9 *vs*. 128±13hpi, *p*=0.03) occurred significantly later than in fresh semen-derived embryos (fresh vs frozen, respectively) (Hickman *et al.*, 2013). Lammers *et al.* (2015) also compared morphokinetic times between the fresh semen group and frozen semen from testicular biopsy in ICSI cycles. They obtained significantly longer times (*p* value <0.05) for the t8 parameters (61.34 (10.8), 55.79 (10.13)) and s2 (9.84 (6.82), 7.98 (6.54)) in the fresh semen group compared to the frozen semen group, respectively. However, they indicated that tSC, tM, tSB and tB cellular events occurred significantly later in the frozen sperm group (Lammers *et al.*, 2015) The results of Hickman *et al.* (2013) and Lammers *et al.* (2015) differ from the results presented in our study, which makes it difficult to draw conclusions. These discrepancies between studies could be due to the large methodological difference between studies, as well as the different origin of the frozen semen (testicular biopsy or ejaculate).

It should be noted that, in our study, the vitrified oocyte group was associated with significantly higher values for t2, t3, t4, t5, tM, tSB. These results are in agreement with the research of Cobo *et al.* (2017), in which significantly longer times (*p* value<0.05) were recorded for the morphokinetic variables t2, t3, t4, t5, tM, tB and s2 in the vitrified oocyte group with respect to the fresh oocyte group. The authors justify these results by indicating that vitrified oocytes initiate cell divisions later, as all cellular processes and metabolic reactions are stopped during vitrification, so that reactivating the machinery can take a long time and could imply additional efforts for the cells, which could imply a higher energetic cost and that processes such as meiotic spindle formation during cell division are delayed (Dumollard *et al.*, 2007; Cobo *et al.*, 2017).

In terms of clinical reproductive outcomes, our data indicated that, although there are lower clinical pregnancy rate, implantation rate, ongoing pregnancy rate, and LBR and higher miscarriage rates for the frozen semen group compared to the fresh semen group, these differences are not significant, so that, the use of frozen semen would not have a negative effect on clinical reproductive outcomes. Some studies have published data similar to those presented (Braga *et al.*, 2015; McCarter *et al.*, 2022; Miller *et al.*, 2022; Punjani *et al.*, 2022), although other investigations present contrary ideas (Subak *et al.*, 1992; Kuczyński *et al.*, 2001; Borges *et al.*, 2007).

Furthermore, most of these studies differ in terms of methodology (artificial insemination (AI), IVF or ICSI), sample size, seminal processing and cryopreservation methods, sperm origin (ejaculate, epididymal or testicular sperm) and sample quality (oligozoospermic, cryptozoospermic or even azoospermic), making it difficult to extrapolate their results with ours.

Subak *et al.* (1992) reported a higher pregnancy rate in the fresh semen group than in the frozen semen group (20.6% *vs*. 9.4%, *p* value<0.03), when IUI was performed with donor sperm. In contrast, Kuczyński *et al.* (2001) found significant differences in the ongoing pregnancy rate between the fresh sperm group (23.7%) and the frozen sperm group (35.2%) (*p* value<0.05), using cryopreserved sperm from oligoasthenozoospermic patients in ICSI cycles.

Furthermore, similar to our data, the authors reported no statistically significant difference in fertilization rate, number of embryos per cycle and morphological quality of cleavage-stage embryos, (*p* value>0.05). Therefore, the authors concluded that cryopreservation of spermatozoa from men with poor sperm quality does not negatively affect fertilization and pregnancy rates (Kuczyński *et al.*, 2001), maintaining the idea of some authors who suggest that properly performed cryopreservation selectively affects defective rather than normal spermatozoa (Ragni *et al.*, 1990; Spanò *et al.*, 1999).

However, Borges *et al.* (2007) found significant differences in the fertilization rate when asthenozoospermic samples were used, with the fertilization rate with fresh sperm being higher than that of the cryopreserved sperm group (75.3% *vs*. 50.5%, *p* value=0.001). However, no significant differences were detected in implantation rates (12.5% *vs*. 15.5%, *p* value=0.702) and pregnancy rates (60.0% *vs*. 50.0%, *p* value=0.741). The authors supported the idea that once the oocyte is fertilized, implantation and pregnancy rates are similar in patients with and without sperm abnormalities (Borges *et al.*, 2007; Di Santo *et al.*, 2012). Braga *et al.* (2015) reported similar data to our study, indicating that embryo quality at the cleavage stage, as well as blastocyst formation, was not influenced by sperm origin when oocyte quality was not taken into account.

A very similar study to ours is that of McCarter *et al.* (2022), where they also found no significant difference in the fertilization rate when using fresh (74.8%) or frozen (68.6%) sperm (*p* value=0.13) in ICSI cycles with donated oocytes and normozoospermic sperm. The adjusted odds of pregnancy (adjusted OR 0.67, 95% CI 0.20, 2.27) and implantation (adjusted OR 0.5, 95% CI 0.12, 2.12) were also not significantly different between the fresh and frozen sperm groups. However, the difference in the delivery rate (fresh 69% fresh, 44% frozen) (*p* value=0.03) and miscarriage rate (5.9% fresh *vs*. 33% frozen) (*p* value=0.013) was significant (McCarter *et al.*, 2022).

Similarly, Miller *et al.* (2022) reported that they found no significant differences in fertilization rate (82.2 (13.2) *vs*. 77.5 (21.7), *p* value=0.37), blastocyst development rate (49.1 (29.1) *vs*. 55.3 (35.8), *p* value=0.52), clinical pregnancy rate (65.4% *vs*. 57.9%, *p* value=0.61) and LBR (61.5% *vs*. 52.6%, *p* value=0.55) when using fresh vs frozen (respectively) good quality ejaculated semen, in ovodonation ICSI cycles with vitrified oocytes. Punjani also found no significant difference in miscarriage rate, LBR and clinical pregnancy after the use of fresh or frozen ejaculated semen in conventional IVF or ICSI cycles for the sperm concentration group ≥5 mil/ml and for the sperm concentration group <5 mil/ml.

Therefore, these 3 recent papers present a methodology and results comparable to those presented in this study, thus describing a clear trend, although not significant, towards better results with fresh sperm, thus arguing for the use of cryopreserved sperm as a reliable alternative to fresh sperm when it is impossible to obtain a fresh sample.

To the best of our knowledge, our work is one of the largest studies to date attempting to evaluate the effect of ejaculated semen cryopreservation on embryo morphokinetics and clinical outcomes. However, it also has some limitations that need to be pointed out. These include the single-center and retrospective nature of the design, which has meant working with some incomplete information for the proportion of viable embryos and for semen quality in some of the cycles. Furthermore, given that the study included a large sample size of data from a prolonged period of time, uncontrolled effects on ovarian stimulation protocols, culture media, cryoprotectants used, and even the effect of different embryologists during transfer could have affected the outcome.

However, the relatively large number of cycles and embryos included in the analysis is sufficient to provide a relevant answer to our study question. In addition, the most generally accepted nomenclature for the annotation of embryo morphokinetics was used, which limits inter-observer variability and theoretically allows the results to be generalized to other users of such systems. On the other hand, it is important to note that significant differences were found in some of the sperm parameters (volume, concentration and motility) between the frozen and fresh semen groups when the descriptive analysis of the cycles included in the study was performed, which seems to indicate that the initially frozen samples had lower sperm quality. This effect may have influenced the results.

## CONCLUSION

This is one of the few studies available to date that analyze the effect of seminal cryopreservation on embryo morphokinetics. The results presented indicate that seminal cryopreservation may affect embryonic morphokinetics, shortening some developmental times. However, no significant differences in embryo development and viable embryo rates were found. The relationship between sperm freezing and embryo cleavage rate or cell cycle duration remains to be elucidated, although there is no clear effect on reproductive outcome.

With all this information, it is once again ratified that seminal cryopreservation is an effective and safe procedure, the use of which can continue to be applied given the many advantages it offers. However, there is a great lack of knowledge about which embryonic mechanisms are affected and in what way.

The outstanding sample size reported in our study has enabled the detection of subtle differences in morphokinetics (very small differences in embryo development) and such effect may be considered as not clinically relevant although alterations in sperm cells by the freezing process may affect embryo cell cycle duration.
